# A novel risk score model for predicting mortality in heart failure with preserved ejection fraction: Insights from the CURE-HF Registry–ApHAC score

**DOI:** 10.1371/journal.pone.0332913

**Published:** 2025-09-23

**Authors:** Takenori Aoki, Sho Suzuki, Masatoshi Minamisawa, Koji Yoshie, Ken Nishikawa, Yukari Okuma, Kazuhiro Kimura, Yasushi Ueki, Yasutaka Oguchi, Tamon Kato, Tatsuya Saigusa, Soichiro Ebisawa, Ayako Okada, Hirohiko Motoki, Koichiro Kuwahara

**Affiliations:** Department of Cardiovascular Medicine, Shinshu University School of Medicine, Matsumoto, Japan; Tehran University of Medical Sciences, IRAN, ISLAMIC REPUBLIC OF

## Abstract

**Aims:**

To create a simple risk score model to predict post-discharge mortality in patients with heart failure with preserved ejection fraction (HFpEF) after treatment for acute decompensated heart failure (HF).

**Methods and results:**

This was a post-hoc analysis from the CURE-HF registry, which was a prospective multi-center cohort study conducted in Japan between July 2014 and March 2019. We included 378 consecutive patients with HFpEF (left ventricular ejection fraction ≥50%) who were followed for two years. The total cohort was randomly divided into two cohorts: the derivation (n = 278) and the validation (n = 100) cohort. The primary endpoint was all-cause mortality within two years. Logistic regression models were used to evaluate the associations between covariates and the primary endpoint, and to produce the risk score model. C-statistics were calculated to assess the discriminative ability of the derived model. The median age was 83 years, and 52% of the patients were female. In total, 97 patients died within two years. No significant differences were observed between the derivation and validation cohorts in baseline characteristics. On multivariable logistic regression analysis with backward stepwise variable selection, the following variables were selected as the components of the risk score model: age, prior HF admission, serum albumin, and creatinine. The model enabled stratification of mortality rates across thirds, and the average predicted versus observed probabilities of death for the low-, intermediate-, and high-risk groups were 8% vs. 9%, 23% vs. 21%, and 46% vs. 49%, respectively. The c-statistic of the derived model in the validation cohorts was 0.764, indicating a good discrimination of the primary endpoint.

**Conclusions:**

We developed a simple risk score model for predicting two-year mortality in patients with HFpEF. This score model may be useful in guiding decisions regarding close follow-up, advanced therapies, or palliative care for these patients.

## Introduction

Heart failure with preserved ejection fraction (HFpEF) is a heterogeneous condition arising from various factors such as hypertension, atrial fibrillation (AF), obesity, and extracardiac comorbidities, and is particularly common in the elderly [1–3]. Owing to the aging of the global population and increased prevalence of coexisting conditions, the number of patients with HFpEF will continue to increase annually. The survival rate of patients hospitalized for HF remains unsatisfactory [4–6], and predicting the prognosis after discharge is crucial for selecting appropriate treatment strategies.

Many risk scores have been developed to assess the risk of adverse events in patients with HF [7–10]. However, it has been reported that prognostic factors differ between heart failure with reduced ejection fraction (HFrEF) and HFpEF, and prognostic prediction models that include patients with HFrEF have limited performance in predicting the prognosis of HFpEF [11,12]. Because HFpEF represents a heterogeneous population and established biomarkers are still lacking, prognostic prediction remains challenging [13]. Recently, a novel risk score for HFpEF was reported [14], which was developed using data from the Dapagliflozin Evaluation to Improve the Lives of Patients With Preserved Ejection Fraction Heart Failure (DELIVER) trial [15], the Angiotensin Receptor Neprilysin Inhibition in Heart Failure With Preserved Ejection Fraction (PARAGON-HF) trial [16], and the Irbesartan in Heart Failure With Preserved Ejection Fraction Study (I-PRESERVE) trial [17]. However, the study was based on data from patients with chronic heart failure with a left ventricular ejection fraction (LVEF) of 40% or higher [14]. Furthermore, the clinical characteristics of patients with HFpEF in Japan differ from those in Western countries: while HFpEF patients in Western countries are often younger and obese, those in Japan tend to be older and lean, highlighting the importance of accumulating data from Asia [13]. A risk score model that accurately predicts the prognosis of patients with HFpEF would be useful for bedside determination.

Given this background, our objective was to create a simple risk score model to predict post-discharge mortality in Japanese patients with HFpEF after treatment for acute decompensated HF (ADHF).

## Methods

### Study design

**Fig 1:**
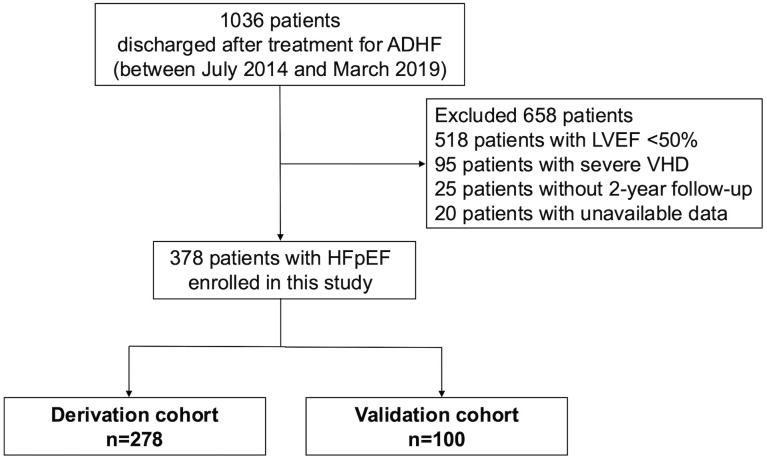
Patient flow chart. ADHF, acute decompensated heart failure; HFpEF, heart failure with preserved ejection fraction; LVEF, left ventricular ejection fraction; VHD, valvular heart disease.

This was a post-hoc analysis from the Clue of Risk Stratification in Elderly Patients with Heart Failure (CURE-HF) registry, which was a prospective multi-center cohort study conducted in Nagano Prefecture, Japan. The CURE-HF registry contains data of 1036 consecutive patients who were hospitalized with a primary diagnosis of ADHF and thereafter discharged after treatment at 15 institutions between July 2014 and March 2019 [18]. Patients aged <20 years, and those with acute coronary syndrome were excluded. In this study, we additionally excluded 518 patients with LVEF <50%, 95 patients with severe valvular heart disease, 25 patients without a two-year follow-up, and 20 patients with unavailable data. Data from the remaining 378 consecutive patients with HFpEF were included in our study (Fig 1). The study was approved by the review board and ethics committee of each participating institution (approval number: 2710). All participants provided written informed consent prior to enrollment. The study was conducted in accordance with the principles of the Declaration of Helsinki and was registered with the University Hospital Medical Information Network (UMIN 000024470).

### Measurements and data collection

Following admission, medical treatment was initiated at the investigator’s discretion. Baseline clinical data, including demographics, medical history, laboratory data, electrocardiograms, and echocardiographic data, were assessed during the compensated state of HF. Follow-up data were acquired from hospital charts and direct contact with patients or their referring physicians. For a thorough assessment of clinical events, additional information was sourced from visits or telephone contact with surviving patients or family members as well as medical records from other hospitals, when necessary, between June 2021 and August 2021. These events were fully anonymized before the analysis by investigators who were blinded to the participants’ identities. Data were accessed on 24/07/2024. The primary endpoint of the study was all-cause death within two years.

### Definitions

Previous hospitalization for HF was determined based on a history of HF diagnosed according to the Framingham criteria [19] and a record of hospitalization for HF. HF was classified according to baseline LVEF in the compensated state before discharge as preserved LVEF (LVEF ≥50%). Cardiac death was defined as death due to HF, death from other cardiac causes, or sudden cardiac death [20]. Hypertension was defined as a systolic blood pressure ≥140 mmHg, diastolic blood pressure ≥90 mmHg, or ongoing therapy for hypertension. Diabetes mellitus was defined as HbA1c ≥ 6.5%, fasting glucose ≥126 mg/dL, random plasma glucose ≥200 mg/dL, or a clinical history of oral hypoglycemic agent and/or insulin use. LVEF was determined by echocardiography using the modified Simpson’s method.

### Statistical analysis

Continuous variables were summarized as mean ± standard deviation if they were normally distributed and as median with interquartile range if they were not normally distributed. Normality was assessed using the Shapiro-Wilk W-test. Baseline characteristics were compared using contingency tables and the χ ^2^ test for categorical variables, the t-test for normally distributed continuous variables, or the Mann-Whitney U test for non-normally distributed continuous variables. The total cohort was randomly divided into two groups using the sample() function in R software: the derivation (n = 278) and the validation (n = 100) cohort (Fig 1). Associations between covariates and the primary endpoint were evaluated using univariable and multivariable logistic regression models with odds ratio (OR)s and 95% confidence interval (CI)s. The following variables, which are clinically considered to be associated with prognosis in HFpEF, were selected as candidates: age, New York Heart Association (NYHA) class, prior HF admission, hemoglobin levels, serum albumin levels, serum creatinine levels, brain natriuretic peptide (BNP) levels, and the presence of atrial fibrillation (AF). A multivariable logistic regression analysis with backward stepwise variable selection was performed to generate the prediction model. Missing data were addressed using multiple imputation by chained equations. Variables selected as components of the prediction model were included in the imputation model. Ten imputed datasets were generated using logistic regression for binary variables and predictive mean matching for continuous variables [21]. Multiple imputation with 10 datasets was considered sufficient given the low missing rate of 2.3% [22,23]. Pooled estimates were calculated according to Rubin’s rules. The Hosmer–Lemeshow test was used to assess the compatibility of the multivariable logistic regression model. C-statistics were calculated to assess the discriminative ability of the derived model. The calibration performance of the model was assessed by comparing the predicted and observed probabilities for the three groups according to the tertile of the risk score. The model was then validated using the validation cohort by assessing its performance, discrimination, and calibration. All tests were 2-sided, and a p-value <0.05 was considered statistically significant. Statistical analyses were performed using SPSS Statistics for Windows version 29 (IBM Corp., Armonk, NY, USA) or R software, version 4.3.1 (R Foundation for Statistical Computing, Vienna, Austria).

## Results

### Patient characteristics

The baseline characteristics of the entire cohort are presented in Table 1. The median age was 83 (interquartile range [IQR], 77–88) years, and 52% (n = 195) of the patients were female. The median LVEF was 62 (IQR, 56–68) %. In total, 97 (26%) patients died (cardiac death, 53; non-cardiac death, 44) within two years. No significant differences were observed between the derivation and validation cohorts. Comparisons between patients who died and those who survived in the derivation and validation cohorts are shown in S1 and S2 Table, respectively. In the derivation cohort, patients who died within two years were older and had lower body mass index (BMI), hemoglobin, and serum albumin levels, higher BNP and serum creatinine levels, and smaller left ventricular sizes. The proportion of patients with prior HF admission and NYHA class III or IV was higher in patients who died than in those who did not.

**Table 1:** Baseline characteristics of patients with heart failure with preserved ejection fraction in the derivation and validation cohorts.

Variable	All patients (n = 378)	Derivation (n = 278)	Validation (n = 100)	p-value
Age (years)	83 [77–88]	83 [77–87]	84 [77–89]	0.564
Female, n (%)	195 (52)	138 (50)	57 (57)	0.207
BMI (kg/m^2^)	21.0 [19.0–24.2]	21.0 [19.0–24.1]	21.2 [19.0–24.8]	0.886
Systolic blood pressure (mmHg)	117 [104–129]	117 [105–128]	116 [102–130]	0.659
Diastolic blood pressure (mmHg)	65 [56–73]	64 [57–73]	65 [55–75]	0.817
NYHA Class III or IV, n (%)	66 (18)	48 (17)	18 (18)	0.868
Prior HF admission, n (%)	99 (26)	70 (25)	29 (29)	0.468
Hypertension, n (%)	281 (74)	201 (72)	80 (80)	0.131
Diabetes mellitus, n (%)	112 (30)	77 (28)	35 (35)	0.170
Atrial fibrillation, n (%)	248 (66)	185 (67)	63 (63)	0.466
**Medication**				
RAS inhibitors, n (%)	254 (67)	185 (67)	69 (69)	0.654
SGLT2 inhibitors, n (%)	15 (4)	13 (5)	2 (2)	0.240
Beta-blockers, n (%)	239 (63)	177 (64)	62 (62)	0.767
MRAs, n (%)	182 (48)	135 (49)	47 (47)	0.789
Loop diuretics, n (%)	313 (83)	229 (82)	84 (84)	0.712
**Laboratory data**				
BNP (pg/mL)	193 [90–377]	195 [94–379]	173 [81–363]	0.941
Hemoglobin (g/dL)	11.3 [10.2–12.8]	11.4 [10.2–12.8]	11.2 [10.1–13.1]	0.719
Albumin (g/dL)	3.3 [3.0–3.7]	3.3 [3.0–3.6]	3.3 [3.1–3.7]	0.246
Creatinine (mg/dL)	1.08 [0.84–1.44]	1.08 [0.84–1.47]	1.10 [0.86–1.39]	0.951
**Echocardiographic data**				
LVEF (%)	62 [56–68]	62 [56–68]	62 [56–68]	0.956
LA diameter (mm)	45 [40–50]	45 [40–50]	45 [40–50]	0.581
LVDd (mm)	45 [41–50]	45 [41–50]	45 [41–49]	0.847
LVDs (mm)	29 [26–33]	29 [26–33]	29 [26–33]	0.908
LV mass index (g/m^2^)	123 [101–149]	121 [100–147]	128 [102–155]	0.246
RWT	0.48 [0.42–0.57]	0.47 [0.41–0.56]	0.49 [0.42–0.58]	0.307
E/e’ (mean)	12.9 [9.9–16.6]	12.8 [9.8–16.3]	13.0 [10.4–17.4]	0.663

Data are presented as mean ± SD, %, or median [interquartile range]. BNP, brain natriuretic peptide; BMI, body mass index; CI, confidence interval; E/e’, the ratio of early diastolic transmitral flow velocity (E) to early diastolic mitral annular tissue velocity (e’); HF, heart failure; LA, left atrial; LV, left ventricular; LVDd, left ventricular diastolic diameter; LVDs, left ventricular systolic diameter; LVEF, left ventricular ejection fraction; MRA, mineralocorticoid receptor antagonist; NYHA, New York Heart Association; RAS, renin angiotensin system; RWT, relative wall thickness; SGLT2, sodium glucose cotransporter 2.

### Constructing a score model to predict two-year mortality

**Fig 2:**
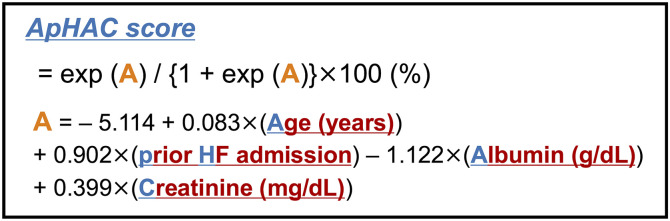
Scheme of the prediction score to estimate the two-year mortality in patients with HFpEF (the ApHAC score). HF, heart failure; HFpEF, heart failure with preserved ejection fraction.

**Fig 3:**
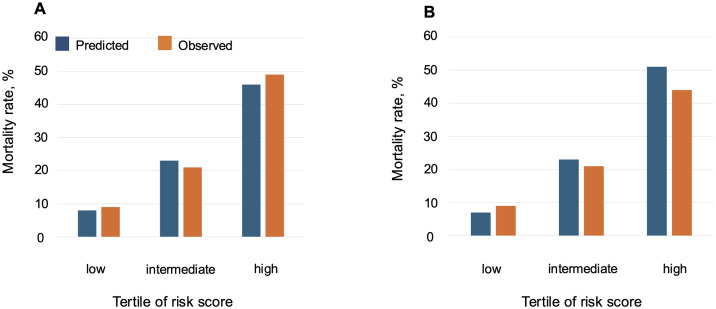
Calibration plots for the model applied to (A) the derivation cohort and (B) the validation cohort.

The results of univariable logistic regression analysis of the primary endpoint in the derivation cohort are shown in Table 2. The following variables were associated with the primary outcome: age (OR, 1.088; 95% CI, 1.045–1.132; p < 0.001), BMI (OR, 0.911; 95% CI, 0.852–0.975; p = 0.007), NYHA class (OR, 2.662; 95% CI, 1.392–5.093; p = 0.003), prior HF admission (OR, 2.198; 95% CI, 1.228–3.934; p = 0.008), hemoglobin levels (OR, 0.771; 95% CI, 0.663–0.897; p < 0.001), serum albumin levels (OR, 0.276; 95% CI, 0.147–0.519; p < 0.001), serum creatine levels (OR, 1.406; 95% CI, 1.034–1.910; p = 0.030), and left ventricular systolic diameter (OR, 0.590; 95% CI, 0.360–0.969; p = 0.037). On multivariable logistic regression analysis, the following variables were selected as the components of the risk score model: age, prior HF admission, serum albumin level, and serum creatinine level (Table 3). Histograms for age, serum albumin, and creatinine levels in the derivation cohort are shown in S1 Fig. The Hosmer–Lemeshow test showed a p-value >0.05 in each step, and the compatibility of the multivariable logistic regression model was good. All 278 patients were included in the final multivariable model after multiple imputation. The rate of missing data ranged from 0% to 3.2% across variables. Based on the results, we developed a novel risk score model to predict two-year mortality in patients with HFpEF (ApHAC score): exp (A)/ {1 + exp (A)} ×100 (%); A = – 5.114 + 0.083×(Age (years)) + 0.902×(prior HF admission) – 1.122×(Albumin (g/dL)) + 0.399×(Creatinine (mg/dL)), (Fig 2). An online calculator was also developed to facilitate estimation of an individual’s risk (https://shinshu-junkanki.jp/aphac-score-calculator/). The model showed good discrimination, with a c-statistic of 0.764 (S2 Fig). The histogram of the ApHAC score in the derivation cohort is shown in S3 Fig. The model enabled stratification of mortality rates across thirds, and the average predicted versus observed probabilities of death for the low-, intermediate-, and high-risk groups were 8% vs. 9%, 23% vs. 21%, and 46% vs. 49%, respectively (Fig 3A).

**Table 2:** Univariable logistic regression analysis predicting death within two years in the derivation cohort.

Variable	Odds ratio (95% CI)	p-value
Age (per year)	1.088 (1.045–1.132)	<0.001
Female	0.912 (0.534–1.557)	0.736
BMI (per kg/m^2^)	0.911 (0.852–0.975)	0.007
Systolic blood pressure (per mmHg)	0.988 (0.973–1.003)	0.116
Diastolic blood pressure (per mmHg)	0.987 (0.964–1.011)	0.288
NYHA Class III or IV	2.662 (1.392–5.093)	0.003
Prior HF admission	2.198 (1.228–3.934)	0.008
Diabetes mellitus	0.663 (0.353–1.244)	0.201
Atrial fibrillation	0.951 (0.537–1.684)	0.863
**Medication**		
RAS inhibitors	1.036 (0.587–1.828)	0.903
SGLT2 inhibitors	1.262 (0.377–4.231)	0.706
Beta-blockers	0.759 (0.439–1.314)	0.325
MRAs	1.513 (0.884–2.591)	0.131
**Laboratory data**		
BNP (per pg/mL)	1.000 (1.000–1.001)	0.391
Hemoglobin (per g/dL)	0.771 (0.663–0.897)	<0.001
Albumin (per g/dL)	0.276 (0.147–0.519)	<0.001
Creatinine (per mg/dL)	1.406 (1.034–1.910)	0.030
**Echocardiographic data**		
LVEF (per %)	1.025 (0.990–1.062)	0.159
LA diameter (per mm)	1.253 (0.938–1.672)	0.127
LVDd (per mm)	0.717 (0.479–1.073)	0.106
LVDs (per mm)	0.590 (0.360–0.969)	0.037
LV mass index (per g/m^2^)	1.001 (0.994–1.007)	0.866
RWT	5.184 (0.719–37.370)	0.103
E/e’ (mean)	1.001 (0.973–1.030)	0.940

Abbreviations are as in Table 1.

**Table 3:** Multivariable logistic regression model predicting death within two years in the derivation cohort.

Variable	Odds ratio (95% CI)	Coefficient	p-value
Age (per year)	1.087 (1.042–1.134)	0.083	<0.001
NYHA Class III or IV	NA	NA	NA
prior HF admission	2.464 (1.298–4.677)	0.902	0.006
BNP (pg/ml)	NA	NA	NA
Hemoglobin (per g/dL)	NA	NA	NA
Albumin (per g/dL)	0.326 (0.161–0.658)	−1.122	0.002
Creatinine (per mg/dL)	1.490 (1.093–2.031)	0.399	0.012

Abbreviations are as in Table 1.

### Validation of the derived ApHAC score

The histogram of the ApHAC score in the validation cohort is shown in S4 Fig. When validated, the ApHAC score showed good discrimination for the primary endpoint (c-statistic = 0.764) (S2 Fig). Fig 3B demonstrates the model’s calibration: in the validation cohort, the average predicted versus observed probabilities of death for the low-, intermediate-, and high-risk groups were 7% vs. 9%, 23% vs. 21%, and 51% vs. 44%, respectively.

## Discussion

This study investigated the predictors of two-year mortality in patients with HFpEF in Japan. We produced a simple risk score model that accurately predicted two-year morality. All included variables were easily measurable by checking their age, prior HF hospitalization, and blood tests. We believe that this risk score could be useful for bedside determination of whether to consider close follow-up, advanced HF therapies, or palliative care consultations.

To date, numerous risk score models investigating the prognosis of HF have been published [7–10,14,24,25]. Classic models were derived by combining HFrEF and HFpEF, despite their distinct clinical characteristics and features [7,9,10]. Recently, it has been demonstrated that prediction scores designed for HFrEF can be challenging to effectively apply to HFpEF [11]. It has been reported that HFpEF and HFrEF differ not only in pathophysiology but also in prognosis, and many previous risk scores have included LVEF itself as a prognostic factor [26]. It has been shown that the HFA-PEFF score, originally used for diagnosing HFpEF [27], is also useful in predicting death and HF readmission in patients with HFpEF [25]. However, this score was originally developed for the purpose of diagnosing HFpEF, requiring detailed echocardiographic data such as global longitudinal strain. Echo stress tests or invasive hemodynamic measurements may also need to be considered. The recently reported risk score model for HFpEF [14] includes patients with an LVEF of 40–49%, which is not classified as HFpEF in the current guidelines [28,29]. HF with mildly reduced ejection fraction (LVEF 41–49%) has been reported to differ from HFpEF in terms of baseline characteristics and underlying pathophysiology [30], and applying this risk score model to HFpEF may result in misclassification or limit its applicability.

In this study, age, history of prior HF admission, serum albumin, and serum creatinine were identified as prognostic factors, all of which have been previously reported as prognostic predictors in past studies [7,14,31]. The results of the present study highlight the importance of nutritional status and cardiorenal syndrome in patients with HFpEF. This study enabled accurate prediction of post-discharge survival rates in HFpEF patients hospitalized for acute HF in Japan. The discriminative ability in the validation cohort was comparable to that in the derivation cohort, and the predictive accuracy remained consistent across patients with both low and high risks. Although NYHA class, BMI, and hemoglobin levels showed significant differences in the univariable logistic regression model, they were not significant in the multivariable logistic model. Regarding NYHA class, 82% of the patients were classified as NYHA I or II after treatment for decompensated HF, and the low number of patients with NYHA III or higher might have influenced the results. With respect to anemia, because it is strongly influenced by age and renal function, particularly in elderly patients, adjustment for these factors may explain why hemoglobin did not remain an independent predictor in this study. In terms of BMI, the relatively low BMI in Japanese patients with HFpEF compared to those in Western countries may have affected the findings. There was no significant difference in BNP levels, an established prognostic predictor of HF, between patients who survived and those who died in the univariable logistic regression analysis. One possible explanation for this result is that BNP levels tend to be lower in HFpEF than in HFrEF patients. The prognostic benefits of angiotensin receptor-neprilysin inhibitor (ARNI) [32] and sodium-glucose cotransporter 2 (SGLT2) inhibitors [15,33], which have been reported to be beneficial in HFpEF, were not observed in this study due to low prescription rates during the enrolment period.

In the present study, we developed a simple risk score model to predict the prognosis of Japanese patients with HFpEF with LVEF ≥50%. The risk score model is a simple tool that can be measured at the bedside and can accurately predicted two-year mortality. We believe that the ApHAC score will be useful to a wide range of healthcare professionals caring for patients with HFpEF. It can be helpful for discussing prognosis with patients and their families, as well as for sharing prognostic information with other healthcare professionals when planning treatment for other conditions, where understanding the expected prognosis might impact therapeutic decisions [34].

This study had several limitations. First, the sample size was small and included only Japanese patients, indicating that Western populations were not evaluated in this study. The performance of our risk score model must be evaluated following their application to people of other ethnicities and countries. Second, this study was performed using data measured at discharge; therefore, application of the model at admission may be limited. However, this investigation aimed to identify patients at high risk of post-discharge mortality; we did not focus on patients who died during hospitalization. Third, although we performed internal validation, further studies are needed to validate the model’s accuracy and investigate its predictive ability in other cohorts. Fourth, the prescription rates of ARNI and SGLT2 inhibitors were low owing to the study period. As therapeutic strategies for HFpEF continue to advance, the low usage of these drugs, which are recommended in current guidelines [28,29], raises concerns about the generalizability of this risk score. Finally, this study did not compare the predictive ability of the developed risk score model with previously reported score models.

In conclusion, we developed a simple risk score model for predicting two-year mortality in Japanese patients with HFpEF. All included variables were easily measurable using age, history of prior HF hospitalization, and routine blood tests. The score model accurately discriminated between patients who survived and those who died within two years after admission for ADHF. This score model may be useful in guiding decisions regarding close follow-up, advanced therapies, or palliative care for these patients. Its generalizability to other populations remains uncertain and requires further investigation.

## Supporting information

S1 Table Baseline characteristics in the derivation cohort. Data are presented as mean ± SD, %, or median [interquartile range]. BNP, brain natriuretic peptide; BMI, body mass index; E/e’, the ratio of early diastolic transmitral flow velocity (E) to early diastolic mitral annular tissue velocity (e’); HF, heart failure; LA, left atrial; LVDd, left ventricular diastolic diameter; LVDs, left ventricular systolic diameter; LVEF, left ventricular ejection fraction; MRA, mineralocorticoid receptor antagonist; NYHA, New York Heart Association; RAS, renin angiotensin system; RWT, relative wall thickness; SGLT2, sodium glucose cotransporter 2.
(XLSX)

S2 Table Baseline characteristics in the validation cohort. Abbreviations are as in S1 Table.
(XLSX)

S1 Fig Histograms of (A) age, (B) serum albumin levels, and (C) serum creatinine levels in the derivation cohort. (TIFF)

S2 Fig Performance of the derived prediction score (ApHAC score) in (A) the derivation cohort and (B) the validation cohort. (TIFF)

S3 Fig Histogram of the ApHAC score in the derivation cohort. (TIFF)

S4 Fig Histogram of the ApHAC score in the validation cohort. (TIFF)

S1 File Original data. (XLSX)
