# Supplementary figures and images for: A novel risk score model for predicting mortality in heart failure with preserved ejection fraction: Insights from the CURE-HF Registry–ApHAC score
Source: PLoS One. 2025 Sep 23;20(9):e0332913. doi: 10.1371/journal.pone.0332913 (PMC12456812; doi:10.1371/journal.pone.0332913)

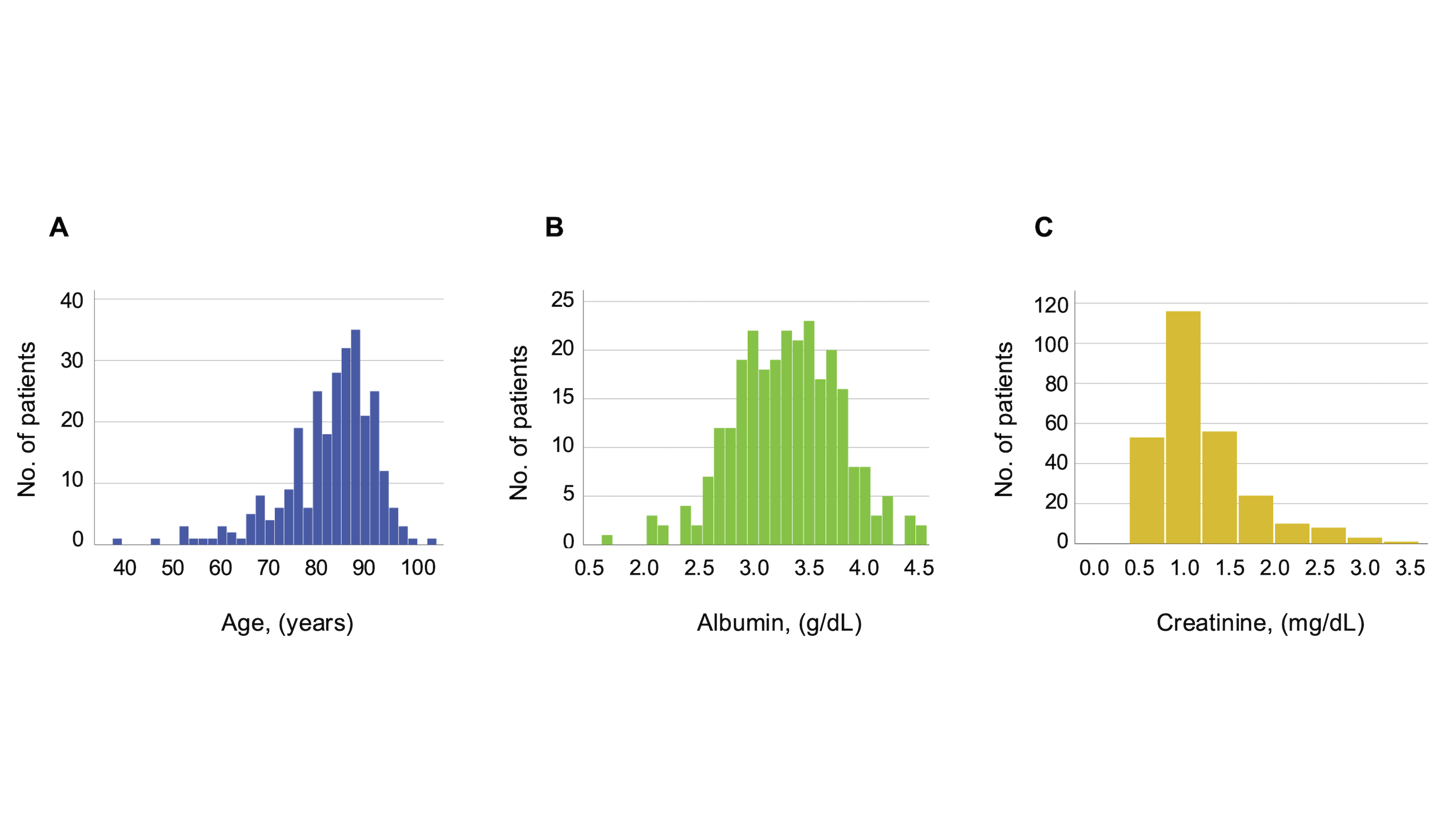

Supplement: S1 Fig — (TIFF) [file pone.0332913.s003.tiff]

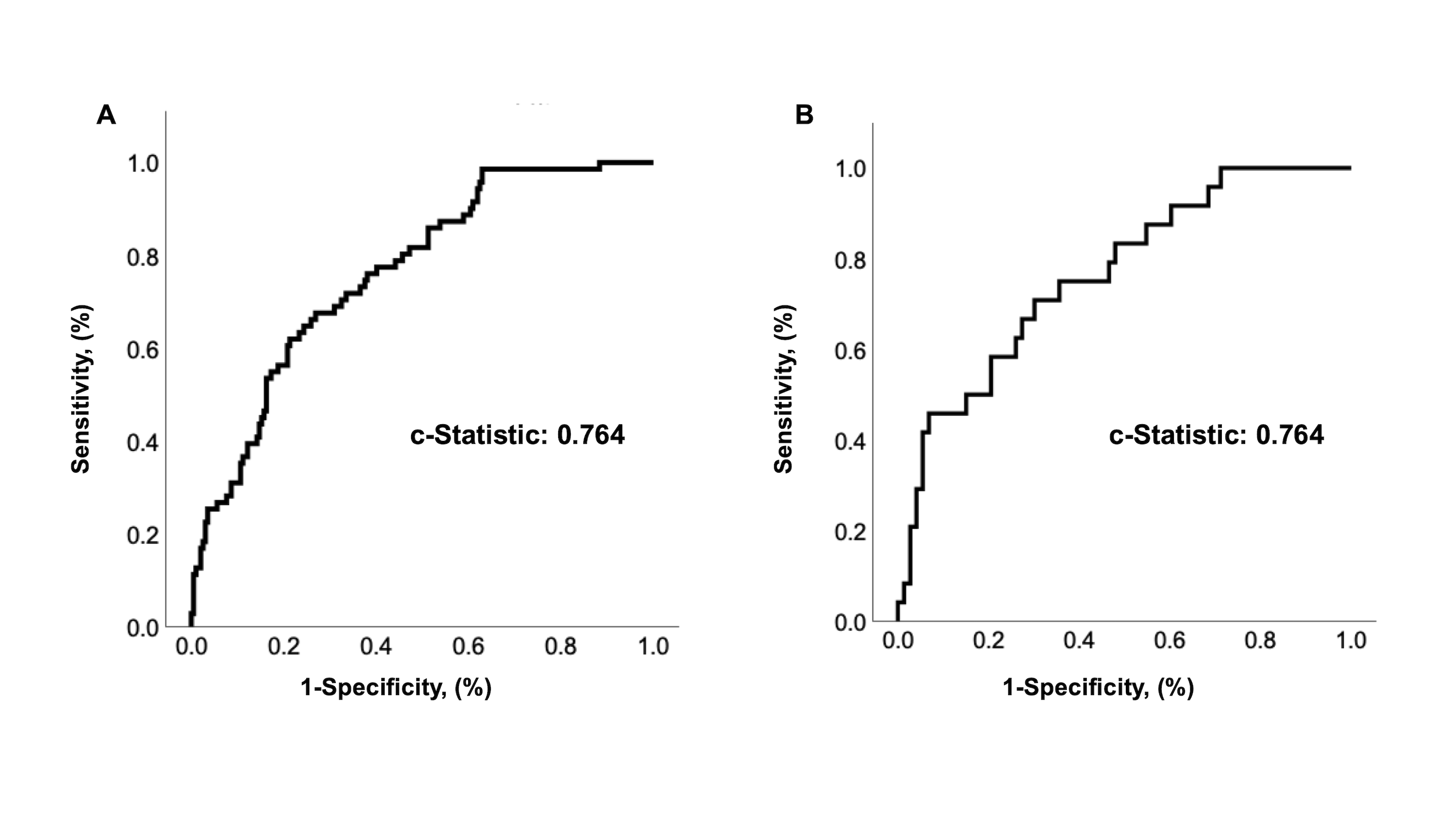

Supplement: S2 Fig — (TIFF) [file pone.0332913.s004.tiff]

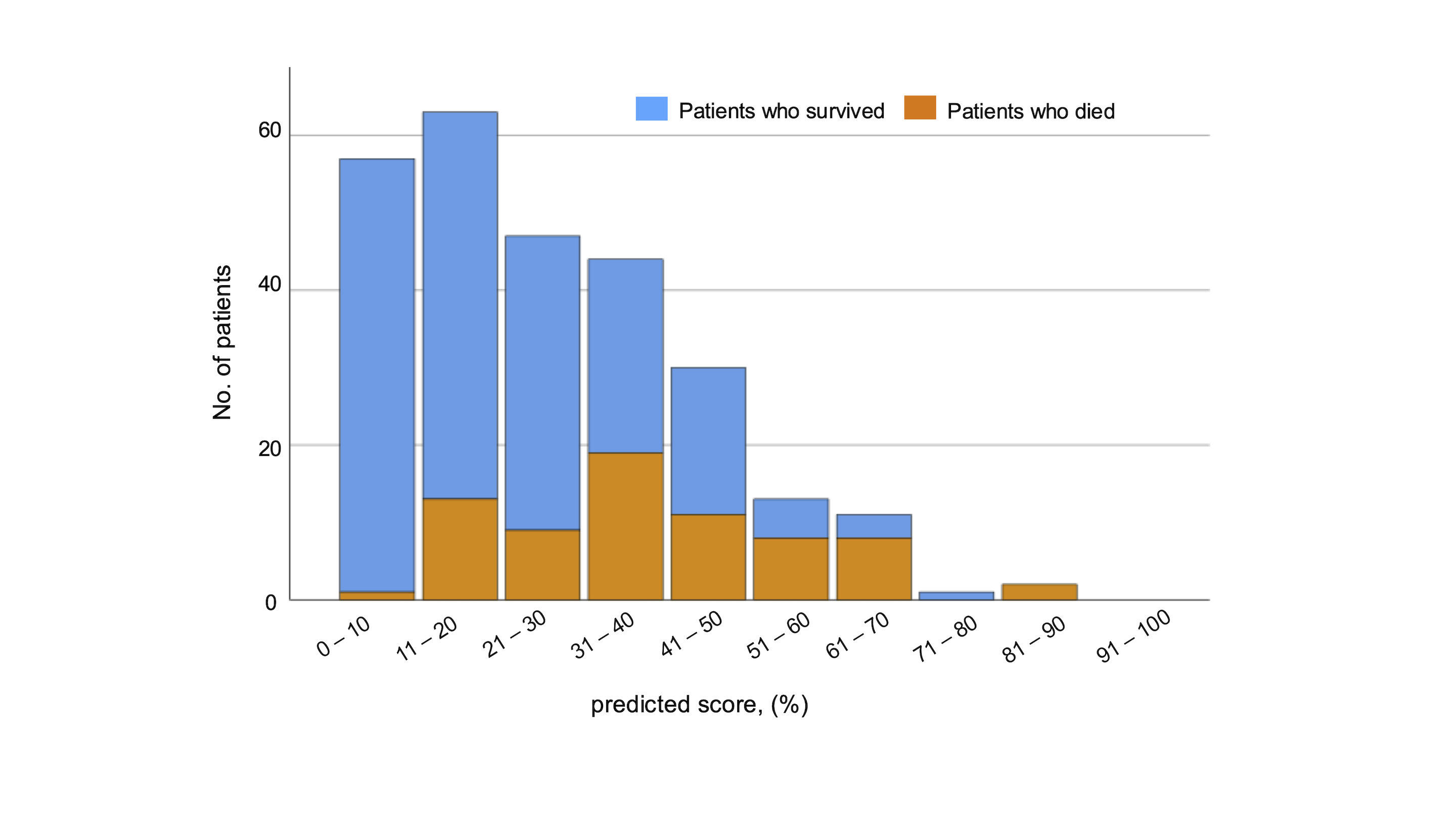

Supplement: S3 Fig — (TIFF) [file pone.0332913.s005.tiff]

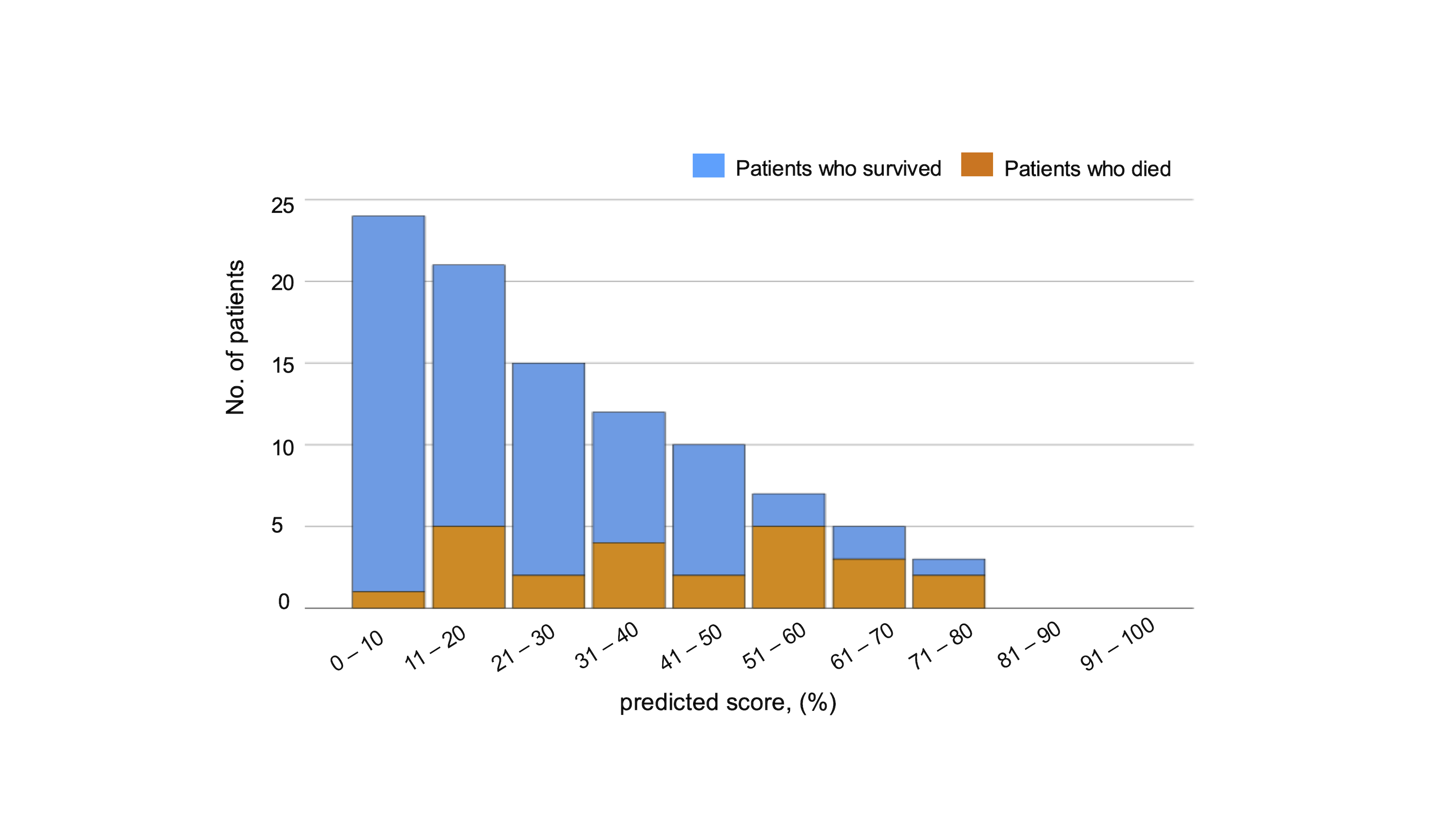

Supplement: S4 Fig — (TIFF) [file pone.0332913.s006.tiff]
